# *FCGR2A* Promoter Methylation and Risks for Intravenous Immunoglobulin Treatment Responses in Kawasaki Disease

**DOI:** 10.1155/2015/564625

**Published:** 2015-05-18

**Authors:** Ho-Chang Kuo, Yu-Wen Hsu, Mei-Shin Wu, Peng Yeong Woon, Henry Sung-Ching Wong, Li-Jen Tsai, Ruo-Kai Lin, Sukhontip Klahan, Kai-Sheng Hsieh, Wei-Chiao Chang

**Affiliations:** ^1^Department of Pediatrics and Kawasaki Disease Center, Kaohsiung Chang Gung Memorial Hospital and Chang Gung University College of Medicine, Kaohsiung 80031, Taiwan; ^2^Department of Clinical Pharmacy, College of Pharmacy, Taipei Medical University, Taipei 11696, Taiwan; ^3^Department of Molecular Biology and Human Genetics, Tzu Chi University, Hualien 97004, Taiwan; ^4^Master Program for Clinical Pharmacogenomics and Pharmacoproteomics, School of Pharmacy, Taipei Medical University, Taipei 11696, Taiwan; ^5^Graduate Institute of Medical Sciences, College of Medicine, Taipei Medical University, Taipei 11696, Taiwan; ^6^Graduate Institute of Pharmacognosy, Taipei Medical University, Taipei 11696, Taiwan; ^7^Graduate Institute of Clinical Medicine, College of Medicine, Kaohsiung Medical University, Kaohsiung 80031, Taiwan; ^8^Department of Pharmacy, Taipei Medical University-Wan Fang Hospital, Taipei 11696, Taiwan

## Abstract

Kawasaki disease (KD) is characterized by pediatric systemic vasculitis of an unknown cause. The low affinity immunoglobulin gamma Fc region receptor II-a (*FCGR2A*) gene was reported to be involved in the susceptibility of KD. DNA methylation is one of the epigenetic mechanisms that control gene expression; thus, we hypothesized that methylation status of CpG islands in *FCGR2A* promoter associates with the susceptibility and therapeutic outcomes of Kawasaki disease. In this study, 36 KD patients and 24 healthy subjects from out-patient clinic were recruited. Eleven potential methylation sites within the targeted promoter region of *FCGR2A* were selected for investigation. We marked the eleven methylation sites from A to K. Our results indicated that methylation at the CpG sites G, H, and J associated with the risk of KD. CpG sites B, C, E, F, H, J, and K were found to associate with the outcomes of IVIG treatment. In addition, CpG sites G, J, and K were predicted as transcription factors binding sites for NF-kB, Myc-Max, and SP2, respectively. Our study reported a significant association among the promoter methylation of *FCGR2A*, susceptibility of KD, and the therapeutic outcomes of IVIG treatment. The methylation levels of CpG sites of *FCGR2A* gene promoter should be an important marker for optimizing IVIG therapy.

## 1. Introduction

Kawasaki disease (KD) is characterized as an acute systemic vasculitic syndrome [1]. It is recognized as a worldwide disease and is thought to be the leading cause of acquired heart disease in children in developed countries [2–4]. Intravenous immunoglobulin (IVIG) is the standard treatment for KD patients. Receiving high dosage of IVIG within seven days after disease onset could reduce the risk of coronary artery damage significantly.

The etiology of Kawasaki disease (KD) remains unclear. However, the epidemiological studies indicated that the prevalence of KD is higher in East Asia than Europe [5]. Uehara et al. suggested that patients with KD family medical history had higher incidence rate than others [6]. Therefore, genetic factor is considered as an important factor in the pathogenesis of KD. Indeed, genome-wide association study (GWAS) and genetics studies have implicated several susceptibility loci for KD [7–15]. Among these studies, the* FCGR2A* was found as one of the important genetic factors. A functional polymorphism of* FCGR2A* elevated the risk of KD [11].

Several lines of evidence have indicated the strong association between methylation status of gene promoter regions and diseases. For example, the CpG methylation pattern of the proximal insulin gene promoter was strongly associated with type 1 diabetes (T1D) in a French T1D cohort [16].* BRCA1* promoter methylation on rs11655505 (c.2265C>T) variants was also reported to associate with sporadic breast cancer [17]. In this study, we designed experiments to investigate the association between methylation status of CpG islands on* FCGR2A* promoter and the clinical outcomes of IVIG therapy in Kawasaki disease.

## 2. Materials and Methods

### 2.1. Patients and Healthy Controls

We recruited 36 KD patients from out-patient clinic and 24 healthy subjects from out-patient clinic in this study (Table 1). KD patients fulfilled the diagnostic criteria as described in our previous studies [12–15]. This study was approved by Institutional Review Board of Kaohsiung Chang Gung Memorial Hospital. Informed consent was obtained from all individuals' parents or guardians before any test was conducted.

### 2.2. Evaluation of IVIG Responsiveness

IVIG-responsiveness was defined as defervescence within 48 hours after initial IVIG and no reappearance of fever (>38°C) for at least 7 days [18]. A standard IVIG treatment was employed and the details of the procedures were described previously [18].

### 2.3. *FCGR2A* Promoter CpG Methylation Analysis

Bisulfite sequencing technology was performed by Pyromark Q24 machine to profile the methylation levels on the* FCGR2A* promotor region within our targeted 500 bps range (1q23.3; chr1:161,474,603-161,475,102). In our target region, eleven CpG methylation loci were predicted by Pyromark Q24 software (Qiagen Inc., Valencia, CA, USA) and we marked the sites according to the order of English alphabet.

### 2.4. Transcription Factors Binding Sites Prediction

JASPAR, an open-access database, was applied to predict the potential transcription factor binding sites (TFBSs) [19]. The motifs of candidate TFBSs were assigned scores by the position weight matrix for the given sequence. The scoring algorithm was reviewed by Stormo GD in year 2000 [20]. Each binding model of the score range has their own uniqueness and cannot be compared directly; therefore, JASPAR normalized these ranges and was displayed as relative matrix score. The method for determining the relative matrix score was described by Lenhard et al. [21]. The database only listed the TFs which have the relative matrix scores above 0.80.

### 2.5. Statistical Analysis

Statistical analysis was performed using SPSS, version 14.0 (SPSS Int., Chicago, Illinois, USA). The difference of methylation levels between patient and control groups was analyzed by the Chi-square (*χ* ^2^) test.

## 3. Results

### 3.1. Characteristics of KD Patients and Normal Controls

60 samples were collected in this study, including 36 KD patients and 24 healthy subjects. The information of age and sex ratio for KD patients and control subjects is shown in Table 1. In the KD group, 13 KD patients were nonresponsive to intravenous immunoglobulin (IVIG) treatment, whereas 22 KD patients were responsive to IVIG therapy and 1 KD patient was without IVIG therapy record.

### 3.2. Methylation Status of* FCGR2A* Promoter Region

Both bisulfite sequencing results and clinical data were combined to investigate the association between KD susceptibility and methylation levels. As shown in Table 2, we found that the increased methylation levels at three of the eleven studied CpG sites associated with KD susceptibility. Among the three CpG sites, a strong association was found between methylation of CpG site J and susceptibility of Kawasaki disease (*P* = 0.0003). In addition, increased methylations at the CpG site G and site H also correlated with the risk of Kawasaki disease (*P* = 0.0038 and *P* = 0.0019, resp.).

### 3.3. Pharmacogenomic Analysis for IVIG Therapy

In the treatment of KD,* IVIG* is one of the major medications. However, some patients were nonresponsive to IVIG (2 g/kg) treatment. We, therefore, further tested whether the promoter methylation status of* FCGR2A* associated with the IVIG treatment outcomes. As shown in Table 3, increased methylation levels on CpG sites B, E, F, H, and J strongly associated with the nonresponsive patients to IVIG (*P* < 0.0001).

### 3.4. Bioinformatics Analysis for Potential Binding Sites

To understand the possible mechanisms of CpG sites, online transcription factor binding site prediction program, JASPAR, was applied to predict the candidate transcription factors (TFs). Among the eight TFs, NF-kB, Myc-Max, and SP2 were significantly predicted to bind to the area that covered at least one base of the CpG methylation sites (Table 4).

## 4. Discussion

Methylation and demethylation of cytosine residues in the promoter regions play a crucial role in many vital biological processes. Disease manifestation due to genomic imprinting such as Angelman syndrome and Prader-Willi syndrome are two of the classical examples of genomic DNA methylation involving on human chromosomal region 15q11-13 [22]. In addition, it has been reported that, in fulminant type 1 diabetes, DNA methylation within the* Foxp3* promoter impaired* TLR9*-induced* FOXP3* expression by attenuating* IRF-7* binding activity [23].* FCGR2A* is the most widely expressed* IgG* receptors in various immune cells including natural killer cells, macrophages, and neutrophils [24]. It has been well documented that the change of methylation levels in the extracellular domain of* FCGR2A* influences the ability of this receptor to bind to human IgG2 [25, 26].

Previous studies have identified* FCGR2A* as the susceptibility gene for KD [8, 9, 11]. A functional polymorphism, encoding an H131R substitution (rs1801274), which is located just outside the CNV region of the Fc portion of IgG, confers elevated risk of KD in various ethnic groups [11]. In addition, Omar et al. transfected HEK cells with these two haplotype variants of* FCGR2A* and studied the functional significance by testing their binding abilities to IgG subclasses [27]. They concluded that* FCGR2A* haplotype variants have different binding ability to IgG1, IgG3, and IgG4. Consistent with previous studies, we found that CpG sites G and J which are corresponding loci to NF-kB and Myc-Max are susceptibility loci of KD. In addition, CpG sites J and K which are corresponding to Myc-Max and SP2 are associated with responsiveness to IVIG treatment. Therefore, these loci might be applied as important markers to evaluate the clinical outcomes of IVIG therapy in KD patients.

There are some limitations in this study. First, we focused on investigating the potential CpG site on* FCGR2A* gene promoter region within a 500 bps region. However, other important CpG sites beyond this region were not included in our study. Therefore, extending the coverage on* FCGR2A* region might be necessary. Second, the SNPs or haplotypes within this promoter region could potentially influence the binding affinity of transcriptional factors such as SP2 or NF-kB; thus, confirmations of KD patients' genotypes are required. Third, the study sample size was relatively small; thus a larger sample size is necessary for a better understanding of this disease.

JASPAR is a conventional tool for predicting TFBSs and TFs. This database employs the optimized position weight matrices (PWM) for the scoring system and provides flexible scanning options to avoid the false-positive results. We also tried to use some other motif prediction programs, such as PROMSCAN [28] and TFSFAC [29]; however, the results were varied from different database. We attribute this to the different algorithms of the scoring system. Therefore, biological validations to confirm the computational prediction will be helpful to improve the study accuracy.

In summary, our study indicated a significant association between the promoter methylation of* FCGR2A*, susceptibility of Kawasaki disease, and therapeutic outcomes of IVIG treatment. The methylation levels of CpG sites of* FCGR2A* gene promoter can be important markers for optimizing IVIG therapy.

## Figures and Tables

**Table 1:** Basal characteristics of patients with Kawasaki disease (KD) and normal controls.

Characteristics	KD patients	Control subjects
Number of subjects	36^†^	24
Gender: male (%)	25 (69.4%)	11 (45.8%)
Age (years)	1.57 ± 1.21	4.91 ± 2.78
Age range	0–5	1–11

^†^The number included 13 IVIG-nonresponsive and 22 IVIG-responsive patients and 1 KD patient without record of using IVIG treatment.

**Table 2:** Means and standard deviations of the percentage of methylation detected at each CpG site in KD patients and normal controls.

Gene	CpG sites	Methylation (%)						*P* value
		KD patients (*N* = 36)			Control subjects (*N* = 24)			
		Mean	SD	95% CI	Mean	SD	95% CI	
*FCGR2A *	A	80.61	5.06	78.90–82.33	78.92	4.42	77.05–80.78	0.1856
	B	62.75	9.51	59.54–65.97	62.31	5.13	60.15–64.48	0.8372
	C	70.16	5.61	68.26–72.06	70.22	6.26	67.57–72.86	0.9711
	D	67.82	5.10	66.10–69.55	66.49	4.69	64.51–68.47	0.3112
	E	61.51	5.87	59.52–63.49	59.84	3.57	58.33–61.35	0.2175
	F	82.16	7.24	79.71–84.61	80.24	5.87	77.76–82.72	0.2852
	G	68.66	5.43	66.82–70.49	64.36	5.35	62.10–66.62	0.0038^**^
	H	43.19	11.83	39.19–47.20	34.92	4.55	33.00–36.84	0.0019^**^
	I	83.74	5.75	81.79–85.68	86.56	6.73	83.72–89.40	0.0874
	J	62.30	8.20	59.52–65.07	55.12	4.98	53.01–57.22	0.0003^***^
	K	67.54	7.19	65.11–69.98	66.86	5.51	64.53–69.18	0.6982

The statistical significance ^**^ *P* < 0.01, ^***^ *P* < 0.001.

TGCAAGCTCTGCCTCC**CG** ^A^GGTTCA**CG** ^B^CCATTCTCCTGCCTCAGCCTCC**CG** ^C^AGTAGCTGGGACTATCTGCCAC**CG** ^D^ **CG** ^E^CC**CG** ^F^ GCTAAATTTTTTTTGTATTATTAGTAGAGA**CG** ^G^GGGTTTCAC**CG** ^H^TGTTAGCCAGGATGGTCT**CG** ^I^ATCTCCTGACCT**CG** ^J^TGATCCACC**CG** ^K^ CCTTGGCCTCCCAAAG.

**Table 3:** Means and standard deviations of the percentage of methylation detected at each CpG site in KD patients responding or not responding to intravenous immunoglobulin treatment.

Gene	CpG sites	Methylation (%)						*P* value
		IVIG-nonresponsive patients (*N* = 13)			IVIG-responsive patients (*N* = 22)			
		Mean	SD	95% CI	Mean	SD	95% CI	
*FCGR2A *	A	80.06	4.51	77.33–82.79	80.94	5.55	78.48–83.40	0.6319
	B	71.40	6.65	67.39–75.42	57.55	7.11	54.40–60.70	<0.0001^***^
	C	72.93	5.64	69.52–76.34	68.40	5.09	66.15–70.66	0.0201^*^
	D	69.46	4.72	66.60–72.31	66.85	5.28	64.50–69.19	0.1516
	E	67.65	4.43	64.97–70.33	57.90	3.03	56.56–59.25	<0.0001^***^
	F	88.34	4.92	85.37–91.31	78.47	5.98	75.82–81.12	<0.0001^***^
	G	68.38	5.71	64.93–71.83	68.95	5.48	66.52–71.38	0.7723
	H	55.32	9.42	49.63–61.02	36.38	6.33	33.57–39.19	<0.0001^***^
	I	83.56	5.59	80.18–86.94	83.48	5.83	80.89–86.06	0.9650
	J	69.26	6.72	65.19–73.32	58.53	6.22	55.77–61.29	<0.0001^***^
	K	71.30	6.92	67.12–75.48	65.27	6.68	62.31–68.24	0.0158^*^

The statistical significance ^*^ *P* < 0.05, ^***^ *P* < 0.0001.

**Table 4:** Possible transcription factor binding sites identified by JASPAR.

Transcription factor	Accession^a^	Score	Relative score^b^	Binding site sequence	Corresponding CpG site^c^
SP1	P08047	7.430	0.87	GCTCTGCCTCC	
NFATC2	Q13469	6.016	0.80	TTCTCCT	
SOX10	NP_008872	6.636	0.90	TTTTGT/CCGTGT	I
NF-kB	NP_003989	9.015	0.85	GGGGTTTCAC	G^*^
YY1	P25490	8.084	0.84	CAGGATGGTCTC	I
USF1	BAA76541	5.780	0.85	ATCTCCTGACC	
Myc-Max	AAH36092	8.449	0.81	GACCTCGTGAT	J^*^
SP2	Q02086	6.046	0.81	CACCCGCCTTGGCCT	K^*^

^*^The corresponding CpG sites reached the statistical significance in Table 2 or Table 3.

^a^Accession number is the NCBI protein accession number.

^b^The relative score is provided by the JASPAR according to the similarity of motif sequence.

^c^The sequence in our study is listed as follows:

TGCAAGCTCTGCCTCC**CG** ^A^GGTTCA**CG** ^B^CCATTCTCCTGCCTCAGCCTCC**CG** ^C^AGTAGCTGGGACTATCTGCCAC**CG** ^D^ **CG** ^E^CC**CG** ^F^ GCTAAATTTTTTTTGTATTATTAGTAGAGA**CG** ^G^GGGTTTCAC**CG** ^H^TGTTAGCCAGGATGGTCT**CG** ^I^ATCTCCTGACCT**CG** ^J^ TGATCCACC**CG** ^K^CCTTGGCCTCCCAAAG.

The sites marked as A–K are the methylation loci.
